# Phytochemistry and Evaluation of the Anti-Inflammatory Activity of the Hydroethanolic Extract of *Virola elongata* (Benth.) Warb. Stem Bark

**DOI:** 10.3390/biology13100776

**Published:** 2024-09-28

**Authors:** Bruna Fioravante Di Serio, Jessica de Araujo Isaias Muller, Marcelo José Dias Silva, Fabiana de Freitas Figueiredo, Domingos Tabajara de Oliveira Martins

**Affiliations:** 1 Post-Graduate Program in Health Sciences, School of Medicine, Federal University of Mato Grosso (UFMT), Cuiabá 78060-900, Brazil; jessicaiamuller@gmail.com (J.d.A.I.M.); fabiana.f.fig@gmail.com (F.d.F.F.); 2 Medicinal Plants and Phytotherapeutics Laboratory, Federal University of Alfenas (UNIFAL), Alfenas 37130-001, Brazil; marcelofarmadias@gmail.com; 3 Pharmacology Division, Department of Basic Health Sciences, School of Medicine, Federal University of Mato Grosso (UFMT), Cuiabá 78060-900, Brazil; domingos.martins@ufmt.br

**Keywords:** *Virola elongata*, inflammation, phytochemistry, leukocytes, cytotoxicity, extract

## Abstract

**Simple Summary:**

*Virola elongata* is popularly used to treat inflammation and in the present study we confirmed the pharmacological potential of this species to reduce the acute inflammation caused by LPS with the reduction of leukocytes infiltration, and topical inflammation induced by croton oil. The extract also showed a reduction of pro-inflammatory cytokines and an increase of anti-inflammatory cytokines *in vivo* and *in vitro*. This study is relevant to develop new treatment strategies for inflammatory diseases based on medicinal plants.

**Abstract:**

Background: Previous studies of the hydroethanolic extract of *Virola elongata* inner stem bark (HEVe) have demonstrated its antioxidant, gastroprotective, and antiulcer properties, but have not evaluated its anti-inflammatory potential. Methods: HEVe was obtained by maceration and phytochemically analyzed. Its systemic anti-inflammatory activity was assessed by its effect on lipopolysaccharide (LPS)-induced peritonitis in mice. HEVe gel (HEgVe) was employed to evaluate topical anti-inflammatory activity by measuring the ear edema resulting from croton-oil-induced dermatitis in mice. A cell viability assay was conducted to determine the non-cytotoxic concentrations of the HEVe. RAW 264.7 cells were stimulated by LPS to determinate cytokine and nitric oxide production. Results: A phytochemical analysis of the HEVe revealed the presence of phenolic acids, neolignans, flavonoids, and monomeric catechins. The oral treatment of acute peritonitis with HEVe reduced the total leukocytes, neutrophils, TNF-α, and IL-1β and elevated IL-10 levels. The application of the HEgVe reduced local edema. The HEVe on the RAW 264.7 cells exhibited no cytotoxicity, and the cells with HEVe displayed reduced TNF-α, IL-1β, and NO levels and increased IL-13 levels. Conclusions: HEVe demonstrated systemic and topical multitarget anti-inflammatory activity, likely due to the combined effects of secondary metabolites. HEVe emerges as a promising herbal remedy for inflammation with minimal cytotoxicity, emphasizing its potential therapeutic significance.

## 1. Introduction

Inflammation is a physiological response inherent to organisms with blood vessels, arising in reaction to damage or pathological stimuli induced by physical, chemical, or biological agents. It serves as a primary defense mechanism and is often marked by hallmark symptoms including swelling, pain, redness, and heat. Inflammation is initiated by vasodilation, fluid extravasation, or the release of growth mediators. In severe cases, inflammation may lead to impaired tissue function [1,2].

The immune response in the inflammatory process can be either innate (nonspecific) or adaptive (specific). The innate response is the initial reaction and acts rapidly against pathogens. Conversely, the adaptive response ensues later, in response to a significant pathogen load, activating lymphocytes that identify and eliminate the invading antigen. This process establishes an immunological memory, enabling the organism to respond more efficiently upon re-exposure [3].

A delicate balance between pro- and anti-inflammatory mediators must be maintained to resolve inflammation. An imbalance can lead to a systemic inflammatory response [4], and the prolonged presence of these stimuli can contribute to various disorders, including cancer, diabetes, obesity, arthritis, neurodegenerative conditions, and cardiovascular diseases [5,6].

Numerous medications can modulate the release of pro- and anti-inflammatory mediators to mitigate the inflammatory response by interfering with crucial pathways in the inflammatory cascade, including cyclooxygenases (COX), mitogen-activated protein kinase (MAPK), and nuclear factor kappa B (NF-kB), among others [7]. Non-steroidal anti-inflammatory drugs (NSAIDs) and steroidal drugs (SAIDs or glucocorticoids) are the most commonly prescribed medications for inflammatory disorders. Additionally, traditional disease-modifying antirheumatic drugs (DMARDs) are administered to manage inflammatory arthritis, connective tissue diseases, and certain types of cancer [8,9,10]. However, prolonged use of these drugs can lead to adverse effects such as weight gain, reduced carbohydrate tolerance, skin reactions, and irritation of the gastrointestinal mucosa [11].

New therapies have targeted inflammatory cytokines and other mediators as effective targets for anti-inflammatory interventions. However, there is a heightened risk of exacerbating opportunistic infections or even triggering cancer [12,13].

Considering the common adverse effects associated with the use of conventional medications, it is imperative to explore phytotherapy. Utilizing medicinal plants offers an effective alternative to traditional therapies, with increased accessibility and reduced cost, which are particularly beneficial for economically disadvantaged populations. Moreover, certain groups of plants contain secondary metabolites with documented pharmacological activity [14].

*Virola elongata* (Benth.) Warb. (*V. elongata*), Myristicaceae, is a tree up to 25 m tall limited to the neotropical flora. It presents reddish exudate on its trunk and creamy exudate on its branches, with simple and narrowly oblong–oblong–elliptic leaves and subglobose fruits [14]. It occurs only in South and Central America, in the countries of Brazil, Colombia, Peru, Ecuador, Venezuela, Panama, Guyana, Honduras, and Nicaragua [15].

Some indigenous tribes in the Amazon have the traditional culture of using the stem bark and the resin of *V. elongata* as hallucinogenic snuff in their ceremonies, and as arrow poison for hunting [16]. Indole alkaloids, the main hallucinogenic constituents of the plant, and non-alkaloidal substances with important pharmacological properties have been identified in the stem bark resin [17].

In Brazil, *V. elongata* is popularly known as “mucuíba”, “bicuíba”, “saconadi”, “ucuúba”, “ucuúba-do-igapó”, and “uiqui”. It is distributed in some states in the north and center-west of the country, in the phytogeographic domain of the Amazon [14,18]. Brazilian traditional medicine utilizes the aqueous infusion and macerated bark of *V. elongata*, prepared with sugar cane brandy (cachaça) or white wine, to address various ailments. These include infections, cancer, high cholesterol, heart, back, or stomach pain, gastric ulcer, uterine inflammation, snake bites, and wound healing [18].

Previous studies investigating the hydroethanolic extract from the stem bark of *V*. *elongata* (HEVe) have revealed its gastroprotective and antioxidant properties. Phytochemical analyses have revealed the presence of phenolic compounds, flavonoids, alkaloids, and phytosterols in both hydroethanolic [19] and diethyl ether extracts of *V. elongata* [16]. HEVe exhibits no cytotoxic effects in CHO-k1 and AGS cells. When acute toxicity was evaluated in mice, a single administration of HEVe (2000 mg/kg orally) did not result in animal mortality. Similarly, treatment with HEVe (300, 600, or 900 mg/kg orally) for 30 days did not induce subacute toxicity in rats [20].

This research was undertaken to assess the anti-inflammatory activity and mechanism of action of HEVe in *in vivo* and *in vitro* models of acute inflammation, given the extensive utilization of stem bark preparations from this species to address inflammation-related, coupled with the lack of studies validating this activity. Additionally, this study aimed to delineate the phytochemical profile of the extract.

## 2. Materials and Methods

### 2.1. Plant Material

The stem bark of *V*. *elongata* in the adult stage was collected in the municipality of Santa Terezinha (10°26′54″ S, 50°37′41″ W), Mato Grosso (MT), Brazil, in August 2015. The research project leading to this article was registered in the National System for the Management of Genetic Heritage and Associated Traditional Knowledge (SisGen no. A070DE0A). Voucher specimens of flowers were deposited in the UFMT herbarium, and assigned specimen number 42580. The latest standardized name of the plant was verified using the Reflora (floradobrasil.jbrj.gov.br (accessed on 26 February 2024)) [21] and World Flora Online (WFO) plant list (https://wfoplantlist.org/ accessed on 26 February 2024) [22].

### 2.2. Drugs, Reagents, and Kits

Ultrapure water was generated using a Milli-Q system (Millipore, Burlington, MA, USA). Dimethylsulfoxide (DMSO), Dulbecco’s Modified Eagle Medium (DMEM), doxorubicin, lipopolysaccharide from *Escherichia coli* (serotype 055:B5), dexamethasone acetate, rezasurin, and croton oil were acquired from Sigma-Aldrich Co. (St Louis, MO, USA). Turk’s solution and Instant Prov^®^ (Quick Panoptic dye) were obtained from New Prov, Brazil. Other reagents included 70% ethanol, trypan blue solution (Dinâmica Química Contemporânea Ltd., Indaiatuba, SP, Brazil), ketamine and xylazine (Syntec, Barueri, SP, Brazil), lecithin (Lecigel^®^, Aqia, Guarulhos, SP, Brazil), and hydroxyethylcellulose (Natrosol^®^, Biovital, São Carlos, SP, Brazil). Streptomycin, penicillin, fetal bovine serum (FBS), TNF-α, IL-1β, IL-10, and IL-13 ELISA detection kits were sourced from Thermo Fisher Scientific, Waltham, MA, USA.

### 2.3. Extract Preparation

The stem bark (2 kg) of *V*. *elongata* was cleansed, cut into 2 cm pieces, and then dried in an oven (model MA035/5 MARCONI, São Paulo, Brazil) with forced air circulation at 35 ± 1 °C until reaching a constant weight, signifying complete moisture elimination. Subsequently, the dried bark was ground using a knife mill with a 40-mesh sieve (Tecnal TE-625, Brazil), and the resulting powder was macerated in a 70% hydroethanolic solution (1:3, *w*/*v*) for 7 days, with twice-daily agitation. Following this period, the material was filtered through filter paper (No. 1), and the macerate obtained was concentrated using a rotary evaporator (model 801, Fisatom, Brazil) under reduced pressure (600 mmHg). Any residual solvent was eliminated by placing the extract in an oven at 38 ± 1 °C for 48 h. The resulting extract was frozen at −86 °C (Indrel^®^ Ultra Freezer IULT 335D, Brazil) and then lyophilized (Scientific LJJ02, Brazil) to obtain the hydroethanolic extract of *V*. *elongata* (HEVe). This extract was identified, stored in amber bottles at −30 °C (Brastemp, Brazil), and dissolved in sterile distilled water before use.

### 2.4. Gel Preparation

The HEVe gel (HEgVe) was formulated at the Formula Certa Manipulation Pharmacy in Cuiabá-MT, under the supervision of pharmacist Silveliane Nogueira Alves de Andrade. Initially, 0.15, 0.45, and 1.35 g of HEVe were weighed. A suitable amount of sterile distilled water was then added to each mass of HEVe for dilution, utilizing a vortex tube shaker (Coleman, Brazil). Subsequently, lecithin (Lecigel^®^, Biovital, Brazil), a powdered agent possessing emulsifying properties, was incorporated in the necessary quantity (q.s.) to enhance the viscosity and bioavailability of the active ingredient. Additionally, hydroxyethylcellulose (Natrosol^®^, Aqia, Brazil), an aqueous and non-ionic gelling agent, was added in sufficient quantity to achieve a total volume of 15 g for the incorporation of the active ingredient, resulting in HEgVe at concentrations of 1%, 3%, and 9%.

### 2.5. Animals

Female Swiss Webster mice (*Mus musculus*) weighing 20 to 25 g were obtained from the central animal house of UFMT. The mice were housed for 5 days prior to experimentation in polypropylene cages under controlled environmental conditions (temperature: 25 ± 1 °C; light/dark cycles: 12 h) and provided with a standard diet (Nuvlab^®^ food, Quimtia, Brazil) and ad libitum access to treated water. Animal experiments were conducted in accordance with the ethical principles in animal experimentation established by the Brazilian College of Animal Experimentation (COBEA) and were approved by the Ethics Committee on the Use of Animals (CEUA) of UFMT under authorization number 23108.01799/2022-47.

### 2.6. Cell Line

RAW 264.7 murine macrophages (BCRJ, code: 0212) were obtained from the cell bank of the Federal University of Rio de Janeiro (BCRJ), Brazil. Upon thawing, the cells were cultured in Dulbecco’s Modified Eagle Medium (DMEM), supplemented with streptomycin (10 mg/mL), penicillin (6 mg/mL), 10% fetal bovine serum (FBS), 1% non-essential amino acids, and 1% glutamine at 37 °C in a 5% CO_2_ atmosphere.

### 2.7. Phytochemical Analysis

The HEVe underwent solid-phase extraction (SPE) clean-up using a C18 cartridge (3 mL, Macherey-Nagel, Chromabond C18, Duren, Germany; particle size 45 μm, diameter and pore size 60 Å). Initially, 10 mg of HEVe were dissolved in 1.5 mL of methanol:water (MeOH:H2O, 80:20, *v*/*v*; MeOH: LC-MS grade, LiChrosolv^®^, and ultrapure MilliQ^®^ EQ 7000 water). The cartridge was preconditioned with 450 µL of MeOH and 450 µL of MeOH:H_2_O (80:20, *v*/*v*). Subsequently, sample of the extract was added to the cartridge and eluted with 1.5 mL of MeOH:H_2_O (80:20, *v*/*v*). The eluate was collected and dried under compressed air at room temperature. The dried sample was then dissolved in methanol to a concentration of 10 ppm and analyzed by mass spectrometry.

#### Direct Flow Infusion Coupled with Electrospray Ionization–Tandem Mass Spectrometry (DFI-ESI-IT-MS^n^) and Ultra High Performance Liquid Chromatography Coupled to Photodiode Array and Electrospray Ionization Mass Detectors (UHPLC-PDA-ESI-MS^n^) Analysis

Direct flow infusion of the samples was conducted on a Thermo Scientific LTQ XL linear ion trap analyzer equipped with an electrospray ionization (ESI) source, operating in negative mode (Thermo, USA) (DFI-ESI-IT-MS^n^). A fused-silica capillary tube was utilized at 300 °C, with a spray voltage of 4.5 kV, capillary voltage of—47 V, and tube lens voltage of—226 V. Nitrogen (with a flow of 50 arbitrary units) and helium (with a flow of 10 arbitrary units) were employed as gases. Initially, a full scan of the mass spectrum was performed to acquire the ions’ data within the established *m*/*z* range of 100–2000. Subsequently, MS^n^ experiments were carried out based on the data obtained in the initial scan, targeting preselected precursor ions, and utilizing a collision energy of 30% and an activation time of 30 ms.

UHPLC-PDA-ESI-IT-MS analysis was conducted using a Thermo Scientific ultra-performance liquid chromatography system, comprising an Accela AS autosampler and a quaternary Accela pump 600 coupled with the LTQ XL linear ion trap mass spectrometer (Thermo, USA). Chromatographic separations were performed on a C18 reversed-phase column (Phenomenex^®^ Luna C18 column, 250 × 4.6 mm, 5 µm). The injection volume was 10 µL, and the flow rate was set at 350 μL/min. The column temperature was maintained at 25 °C, and UV detection was conducted at 254 and 354 nm. Sample elution was achieved using a linear gradient of water acidified with 0.1% formic acid (solvent A) and acetonitrile (solvent B), ranging from 5 to 100% (B) over 60 min. Data acquisition and processing were performed using Xcalibur™ version 1.3 (Thermo Finigan, USA) software.

### 2.8. In Vivo Assays

The doses of HEVe chosen for this trial were based on the work of Almeida et al. (2019) [19], which used doses of 100, 300, and 900 mg/kg p.o. in antiulcer activity trials; these doses were equally effective in most trials. Therefore, the lowest dose (100 mg/kg) with antiulcer activity was selected and, from this, lower (5 mg/kg) and intermediate (25 mg/kg) doses were determined to evaluate the anti-inflammatory effect of HEVe.

#### 2.8.1. Lipopolysaccharide-Induced Peritonitis

The peritonitis model [23] was employed to assess the anti-inflammatory potential of HEVe. Following a 12 h fast for food and a 1 h fast for water, female mice (n = 8/group) were pretreated via orogastric gavage (p.o.), with either vehicle (distilled water, 0.1 mL/10 g b.w.), HEVe (at doses of 5, 25, and 100 mg/kg), or dexamethasone (0.5 mg/kg). The naive group received distilled water (0.1 mL/10 g) via the same route. Subsequently, lipopolysaccharide (LPS) from *Escherichia coli* serotype 055:B5 (250 ng/well/0.2 mL in sterile 0.9% saline solution) was administered intraperitoneally (i.p.), except for the naive group, which received an equal volume of 0.9% sterile saline solution i.p. Six hours post-inflammatory stimulus, mice were euthanized with an intraperitoneal (i.p.) overdose of ketamine and xylazine, followed by injection of 3 mL of ice-cold 0.1% phosphate-buffered saline (PBS) containing 1% bovine serum albumin. Peritoneal lavage was collected and utilized for total and differential leukocyte counts. Aliquots of the peritoneal lavage were centrifuged at 3100× *g* for 10 min, and the supernatant was stored at -86 ºC for subsequent determination of cytokines [24].

##### Total and Differential Leukocyte Count

Peritoneal lavage was utilized for both total and differential leukocyte cell counts. The total leukocyte count was determined by diluting 20 µL of the lavage in 380 µL of Turk’s solution (1:19 *v*/*v*), and the count was performed using a Neubauer chamber under an optical microscope at 100× magnification. For the differential count, slides were prepared by smearing 20 µL of peritoneal lavage and staining with Instant Prov^®^ dye (New Prov, Brazil). A total of 100 cells per slide were counted under the microscope, and they were classified as polymorphonuclear (PMN) or mononuclear (MN) based on conventional morphological criteria. The results were expressed as the number of cells × 10^6^.

##### *In Vivo* Cytokine Assay

The concentrations of TNF-α, IL-1β, and IL-10 in the peritoneal lavage of mice were assessed using commercially available enzyme-linked immunosorbent assay (ELISA) kits, following the manufacturer’s instructions (Thermo Fisher Scientific, USA). Absorbance was measured at 540 nm using a microplate reader spectrophotometer (Thermo Fisher Scientific), and the results were graphically expressed in pg/mL.

#### 2.8.2. Croton-Oil-Induced Topical Dermatitis

Topical dermatitis [25] was used to evaluate the potential local anti-inflammatory effect of HEgVe. The inner surfaces of both ears of mice (*n* = 8/group) were topically treated with either vehicle (Natrosol^®^ gel), HEgVe (at concentrations of 1%, 3%, and 9%), or 0.25% dexamethasone (0.05 mg/ear/20 µL). After 25 min, 20 µL of croton oil (2.5% in acetone:water, 7:3 *v*/*v*) were applied to the left ear of each animal, while 20 µL of acetone:water (7:3 *v*/*v*) were applied to the right ear, serving as a control. Six hours after the administration of the inflammatory stimulus, the animals were euthanized with an anesthetic overdose of xylazine (30 mg/kg) and ketamine (300 mg/kg) intraperitoneally (i.p.), and both ears were excised at the base and weighed individually. Topical inflammation was assessed by the local edema formed, calculated by the differences in weight (Δ mg) between the left (inflamed) and right (uninflamed) ear.

### 2.9. In Vitro Assays

#### 2.9.1. Cell Viability Assay

This assay was conducted to determine non-cytotoxic concentrations of HEVe for use in *in vitro* pharmacological assays. The Alamar Blue^®^ (resazurin) test, based on Nakayama’s colorimetric method [26], was employed to assess cell viability. RAW 264.7 cells, cultured in DMEM medium supplemented with 10% fetal bovine serum (FBS) and maintained at 37 °C and 5% CO_2_, were seeded at a density of 2 × 10^4^ cells/mL in duplicate in a 96-well plate. The wells were filled with DMEM culture medium (sterility control) supplemented with 10% FBS and, after 24 h, treated with various concentrations of HEVe (ranging from 6.25 to 400 µg/mL) and doxorubicin (0.0058–58 µg/mL) as a positive control. After 24, 48, and 72 h, the treatments were removed, and rezasurin (10% *v*/*v*) in DMEM medium was added to the plates. The plates were then incubated for 6 h at 37 °C and 5% CO_2_ for reading. The percentages of resazurin and its reduced form (resorufin) were calculated, and the results were expressed as the 50% inhibitory concentration (IC_50_), which represents the concentration of the drug that inhibits 50% of the cells. The samples were read using a microplate reader at absorbances of 540 and 620 nm. IC_50_ values of less than 4 µg/mL and 30 µg/mL were considered cytotoxic for pure and impure substances, respectively [27].

#### 2.9.2. Indirect Determination of Nitric Oxide Production

NO production was determined by the quantity of nitrite (NO_2_^−^), which was calculated using the colorimetric method based on the Griess reaction [28]. RAW 264.7 cells were treated with three concentrations of HEVe (1, 5, and 20 µg/mL) and Nω-Nitro-L-arginine-methyl-ester (L-NAME, 10 mM). Cells were also maintained with only the culture medium and no phenol red (for basal control). After 1 h, they were stimulated with LPS (1 µg/mL), except for the basal group. After 24 h in the oven, the supernatant was collected (100 µL) after 30 min in the centrifuge at 15,000× *g* and incubated with an equal volume of Griess reagent (1% sulfanilamide, 0.1% N-(1-naphthyl)-ethylenediamine dihydrochloride, 2.5% H_3_PO_4_) in another 96-well culture plate for 15 min at room temperature. The absorbance (540 nm) was measured in an automatic microplate reader, and nitrite concentrations were calculated by extrapolation to a sodium nitrite (NaNO_2_) standard curve, with data expressed in μM.

#### 2.9.3. *In Vitro* Cytokine Assay

Initially, RAW 264.7 cells were seeded at a density of 1.5 × 10^5^ cells/well in 24-well plates and incubated in DMEM culture medium supplemented with fetal bovine serum (10%) overnight at 37 °C in a 5% CO_2_ atmosphere. Following a 1 h treatment with HEVe (1, 5, and 20 µg/mL) and dexamethasone (10 mM), cells were stimulated with LPS (1 µg/mL). A control group was maintained with only the culture medium (for basal control). After 24 h of incubation, the supernatant from the stimulated cells was collected, centrifuged at 15,000× *g* for 30 min, and stored at −86 °C for subsequent cytokine quantification. TNF-α, IL-1β, and IL-13 concentrations in RAW 264.7 cells stimulated by LPS (1 µg/mL) were determined using commercially available ELISA kits following the manufacturer’s instructions (Thermo Fisher Scientific). Absorbance readings were taken at 540 nm using a spectrophotometric microplate reader (Thermo Fisher Scientific, USA), and the results were graphically expressed in pg/mL.

### 2.10. Data Analysis

Parametric data were presented as mean ± standard error (S.E.) and analyzed using the one-way analysis of variance (ANOVA) technique for group comparison. Post hoc analysis was performed using the Student–Newman–Keuls multiple comparison test whenever statistical significance was observed. A *p*-value of less than 0.05 was considered statistically significant. The IC_50_ value was determined by linear regression, correlating the percentage of inhibition with the logarithm of the concentrations tested, with a confidence level of 99% (*p* < 0.01) for the curve. *In vitro* assay results that did not involve statistical analysis were expressed as the mean ± standard error (S.E.) of two or three independent experiments. Data tabulation and analysis were performed using GraphPad Prism software (version 5.01, USA).

## 3. Results

### 3.1. Phytochemical Analysis of HEVe

**Figure 1:**
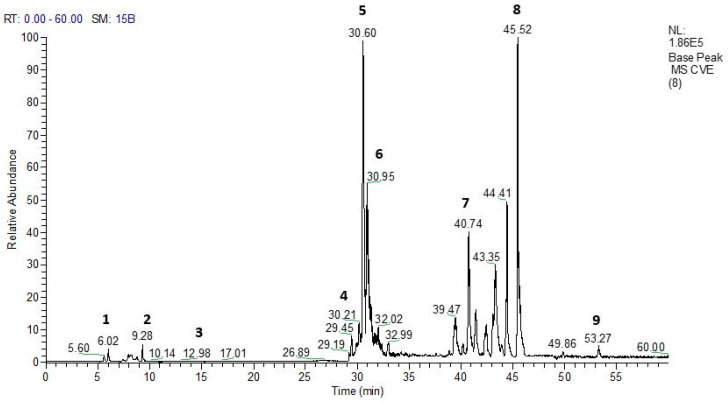
UHPLC-PDA-ESI-IT-MS^n^ analysis of the hydroethanolic extract of *Virola elongata* inner stem bark (HEVe) was conducted, with detection using base peak ion (BPI). With a signal correspondence value of 1.86 × 10^5^. The highlighted peaks represent some substances highlighted in Table 1.

The mass spectrometry analysis of the phytochemical profile of HEVe revealed the presence of phenolic acids, neolignans, flavonoids, and monomeric catechins (Figure 1). Compound identification was achieved by comparing the fragmentation patterns with data from the literature [19,29,30,31]. The UHPLC-MS chromatogram of the extract revealed several compounds: (1) quinic acid, (2) resveratrol, (3) 3′,4′-dimethoxy-3,4-methylenedioxy-6 7′,8 8′-neolignan, (4) quercetin, (5) catechin dimer, (6) diglycosyl-flavonoid (C-glycoside), (7) catechin trimer, (8) catechin tetramer, and (9) catechin pentamer (see attached Supplementary Materials Figures S1–S9) Table 1 details these compounds.

This compounds analysis enhances our understanding of the pharmacological activities of HEVe in the anti-inflammatory models discussed below.

### 3.2. In Vivo Anti-Inflammatory Activity

#### 3.2.1. Effect of HEVe on LPS-Induced Peritonitis

##### Effect of HEVe on Total and Differential Leukocyte Counts

**Figure 2:**
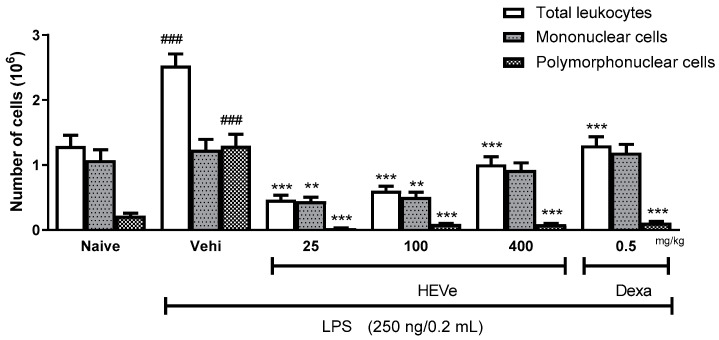
Effect of oral administration of vehicle (Vehi 0.1 mL/10 g distilled water), hydroethanolic extract from the inner stem bark of *Virola elongata* (HEVe 5, 25, and 100 mg/kg), and dexamethasone (Dexa 0.5 mg/kg) on total cells, mononuclear (MN) cells, and polymorphonuclear (PMN) cells present in the intraperitoneal fluid of mice with LPS-induced peritonitis (250 ng/0.2 mL/well). Each column represents the mean ± SE of eight animals/group. One-way ANOVA, followed by the Student–Newman–Keuls test, ^###^ *p* < 0.001 vs. naive, ** *p* < 0.01, and *** p < 0.001 vs. vehi.

The injection of LPS into the mice in the vehicle group resulted in a 95.7% increase (*p* < 0.001) in the total number of leukocytes in the peritoneal lavage compared to the naive group. The oral administration of HEVe at doses of 5, 25, and 100 mg/kg reduced (*p* < 0.001) the total leukocyte count by 69.2%, 81.6%, and 76.3%, respectively. The treatment with dexamethasone (0.5 mg/kg) led to a 48.6% reduction (*p* < 0.001) in the total leukocyte count compared to the vehicle group (Figure 2).

The injection of LPS into the mice in the vehicle group did not induce changes in the number of mononuclear (MN) cells compared to the naive group. However, the oral administration of HEVe at doses of 25 and 100 mg/kg reduced the number of MN cells by 64.3% and 58.0% (*p* < 0.01), respectively, compared to the vehicle group (Figure 2).

The polymorphonuclear (PMN) cell count in the vehicle group increased by 494.9% (*p* < 0.001) compared to the naive group. The treatment with HEVe (5, 25, and 100 mg/kg) resulted in reductions of 94.8%, 98.0%, and 93.0% (*p* < 0.001), respectively, in the number of PMNs compared to the vehicle group, while the treatment with dexamethasone (0.5 mg/kg) led to a 91.4% reduction (*p* < 0.001) (Figure 2).

##### Effect of HEVe on Cytokine Production

**Figure 3:**
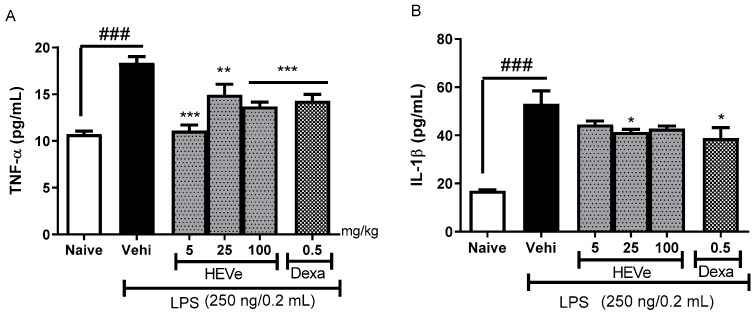
Effect of oral administration of the vehicle (Vehi 0.1 mL/10 g of distilled water), hydroethanolic extract from the inner stem bark of *Virola elongata* (HEVe 5, 25, and 100 mg/kg), and dexamethasone (Dexa 0.5 mg/kg) on the concentrations of (**A**) tumor necrosis factor-alpha (TNF-α) and (**B**) interleukin-1 beta (IL-1β) in the peritoneal lavage of mice with peritonitis induced by intraperitoneal injection of LPS (250 ng/well/0.2 mL). The naive group received distilled water (0.1 mL/10 g p.o.) and 0.9% saline (0.2 mL/well i.p.). Each column represents the mean ± SE of eight animals/group. One-way ANOVA, followed by the Student–Newman–Keuls test, * *p* < 0.05 vs. Vehi, ** *p* < 0.01 vs. Vehi, *** *p* < 0.001 vs. Vehi, ^###^ *p* < 0.001 vs. naive.

In the animals with LPS-induced peritonitis, there was a 71.1% increase (*p* < 0.001) in the levels of the pro-inflammatory cytokine TNF-α compared to the naive group. The treatment with HEVe at doses of 5, 25, and 100 mg/kg reduced TNF-α levels in the peritoneal lavage by 39.6% (*p* < 0.001), 18.6%, and 25.5% (*p* < 0.01), respectively, compared to the vehicle group. Dexamethasone (0.5 mg/kg), used as a positive control, reduced TNF-α levels by 22.2% compared to the vehicle (*p* < 0.001) (Figure 3A).

The levels of the pro-inflammatory cytokine IL-1β increased by 213.6% (*p* < 0.001) in the peritoneal lavage of the peritonitis group compared to the naive group. The treatment with HEVe at a dose of 25 mg/kg decreased IL-1β levels by 22.1% (*p* < 0.05) compared to the vehicle group, while dexamethasone (0.5 mg/kg), a positive control, reduced the IL-1β levels by 26.7% (*p* < 0.05) (Figure 3B).

**Figure 4:**
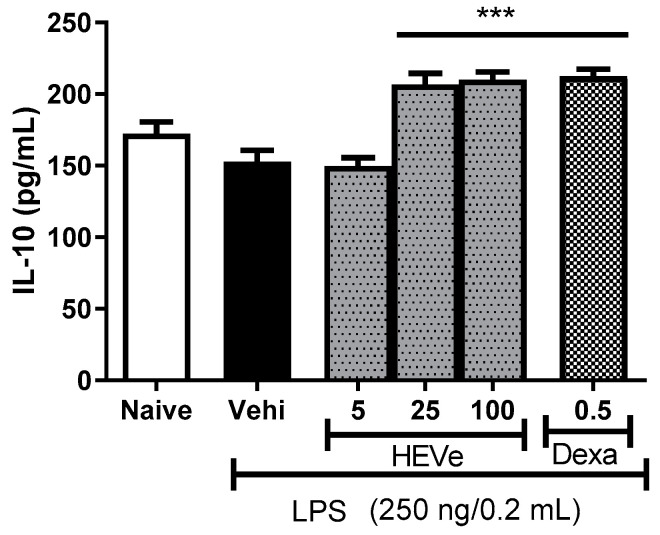
Effect of oral administration of the vehicle (Vehi 0.1 mL/10 g of distilled water), hydroethanolic extract from the inner stem bark of *Virola elongata* (HEVe 5, 25, and 100 mg/kg), and dexamethasone (Dexa 0.5 mg/kg), on the concentration of interleukin 10 (IL-10) in the peritoneal lavage of mice with peritonitis induced by intraperitoneal injection of LPS (250 ng/well/0.2 mL). The naive group received distilled water (0.1 mL/10 g p.o.) and 0.9% saline (0.2 mL/well i.p.). Each column represents the mean ± SE of eight animals/group. One-way ANOVA, followed by the Student–Newman–Keuls test, *** *p* < 0.001 vs. vehi.

The animals with LPS-induced peritonitis did not demonstrate a significant difference in the determination of the anti-inflammatory cytokine IL-10 compared to the naive group. Treatment with HEVe at doses of 25 and 100 mg/kg increased the levels of this cytokine in peritoneal lavage by 35.5% and 37.7%, respectively (*p* < 0.001), compared to the vehicle group. Dexamethasone (0.5 mg/kg), a positive control, increased IL-10 levels by 39.1% (*p* < 0.001) (Figure 4).

#### 3.2.2. Effect of HEVe on Topical Inflammation

**Figure 5:**
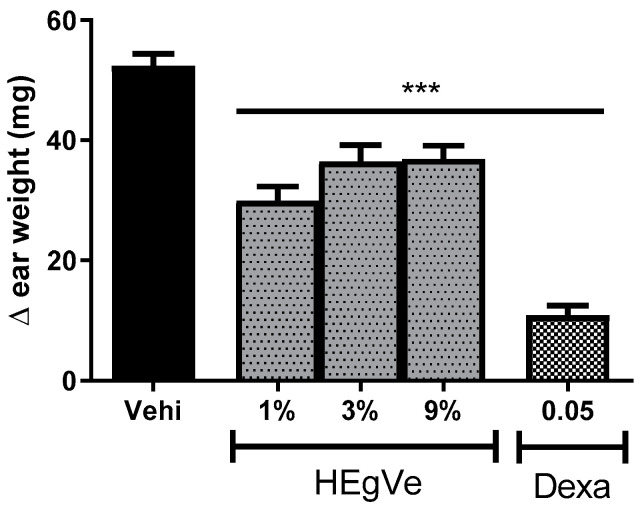
Difference in weight of the ears of female mice after topical treatment with the vehicle (Vehi 20 µL of 2.5% croton oil in acetone:water (70:30)), hydroethanolic extract gel from the inner stem bark of *Virola elongata* (HEgVe 1, 3, and 9%), and dexamethasone (Dexa 0.05 mg/ear/20 µL). Each column represents the mean ± SE of eight animals/group. One-way ANOVA, followed by the Student–Newman–Keuls test, *** *p* < 0.001 vs. vehi.

The topical treatment with HEVe gel (1, 3, and 9%) reduced croton-oil-induced ear edema by 43.0%, 30.4%, and 29.5%, respectively (*p* < 0.001), compared to the vehicle group. The application of dexamethasone at 0.05 mg/ear/20 µL (0.25%) reduced local edema by 79.27% (*p* < 0.001) (Figure 5).

### 3.3. In Vitro Anti-Inflammatory Activity

#### 3.3.1. Effect of HEVe on Cell Viability

**Figure 6:**
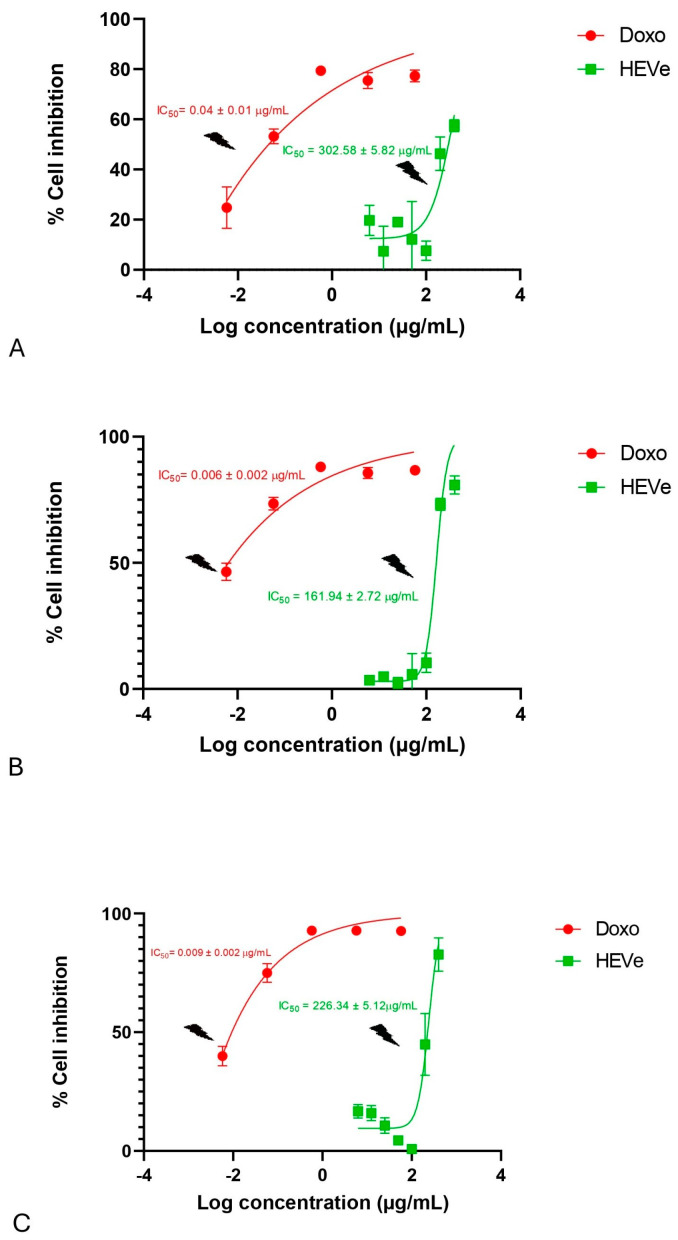
Cytotoxicity assessment of the hydroethanolic extract from the bark of the stem of *Virola elongata* (HEVe 6.25–400 µg/mL) and doxorubicin (Doxo 0.0058–58 µg/mL in RAW 264.7 cells stimulated with lipopolysaccharide (LPS 1 µg/mL) for 24 (**A**), 48 (**B**), and 72 h (**C**). Inhibitory concentration at 50% (IC_50_ ± S.E.). Nonlinear regression analysis (curve fitting).

The treatment of the RAW 264.7 cells with HEVe (6.25–400 µg/mL) did not induce cytotoxicity, demonstrating IC_50_ values of 302.58 ± 5.82 μg/mL at 24 h (Figure 6A), 161.94 ± 2.72 μg/mL at 48 h (Figure 6B), and 226.34 ± 5.12 μg/mL at 72 h (Figure 6C). However, doxorubicin (0.0058–58 µg/mL), the standard drug used in this trial, exhibited toxicity in these cells, with IC_50_ values of 0.04 ± 0.01 μg/mL at 24 h (Figure 6A), 0.006 ± 0.002 μg/mL at 48 h (Figure 6B), and 0.009 ± 0.002 μg/mL at 72 h (Figure 6C). 

#### 3.3.2. Effect of HEVe on Nitric Oxide Production

**Figure 7:**
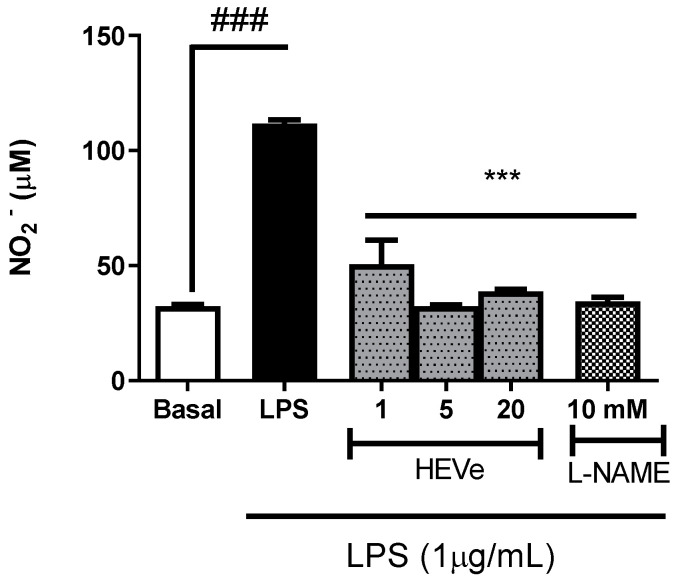
Effect of *in vitro* treatment with hydroethanolic extract from the bark of the stem of *Virola elongata* (HEVe 1, 5, and 20 µg/mL) and Nω-Nitro-L-arginine-methyl-ester (L-NAME 10 mM) on nitric oxide (NO) concentrations in RAW 264.7 cells stimulated with lipopolysaccharide (LPS 1 μg/mL) for 24 h. Cells in the basal group were treated with DMEM culture medium with 10% fetal bovine serum (FBS). One-way ANOVA, followed by the Student–Newman–Keuls test, ^###^ *p* < 0.001 vs. Basal, *** *p* < 0.001 vs. LPS.

The stimulation of the RAW 264.7 cells with LPS (1 μg/mL) resulted in a 245.2% increase (*p* < 0.001) in the level of NO in the cell supernatant compared to the basal group. The treatment with HEVe at concentrations of 1, 5, and 20 μg/mL reduced NO levels by 54.8%, 71.0%, and 65.3%, respectively (*p* < 0.001), compared to the vehicle group. The treatment with L-NAME (10 mM) led to a reduction of 69.2% (*p* < 0.001) compared to the vehicle group (Figure 7).

#### 3.3.3. Effect of HEVe on the Production of Cytokines

**Figure 8:**
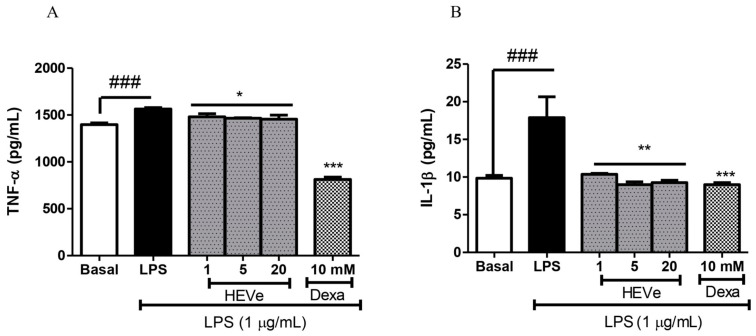
Effect of *in vitro* treatment with hydroethanolic extract from the inner stem bark of *Virola elongata* (HEVe 1, 5, and 20 µg/mL) and dexamethasone (Dexa 10 mM) on the concentration of the cytokines (**A**) TNF-α and (**B**) IL-1β in LPS (1 µg/mL)-stimulated RAW 264.7 cells. Cells in the basal group were treated only with DMEM culture medium with 10% fetal bovine serum (FBS). One-way ANOVA, followed by the Student–Newman–Keuls test, * *p* < 0.05 vs. LPS, ** *p* < 0.01 vs. LPS, *** *p* < 0.001 vs. LPS, ^###^ *p* < 0.001 vs. Basal.

The levels of the pro-inflammatory cytokine TNF-α in the RAW 264.7 cells increased by 11.87% (*p* < 0.001) in the LPS group (1 µg/mL) compared to the basal group. The treatment with HEVe at 1, 5, and 20 µg/mL concentrations reduced the TNF-α levels by 5.3%, 6.3%, and 7.0%, respectively (*p* < 0.05), compared to the cells treated only with LPS. Dexamethasone (10 mM), the positive control, reduced TNF-α levels by 48.09% compared to the LPS-treated group (*p* < 0.001) (Figure 8A).

The levels of the cytokine IL-1β by 81.8% (*p* < 0.001) increased in the LPS group compared to the basal group. The treatment with HEVe at 1, 5, and 20 µg/mL concentrations reduced IL-1β levels by 42.0%, 49.7%, and 48.3%, respectively (*p* < 0.01), compared to the group treated only with LPS. Dexamethasone (10 mM), the positive control, reduced IL-1β levels by 49.8% compared to LPS (*p* < 0.001) (Figure 8B).

**Figure 9:**
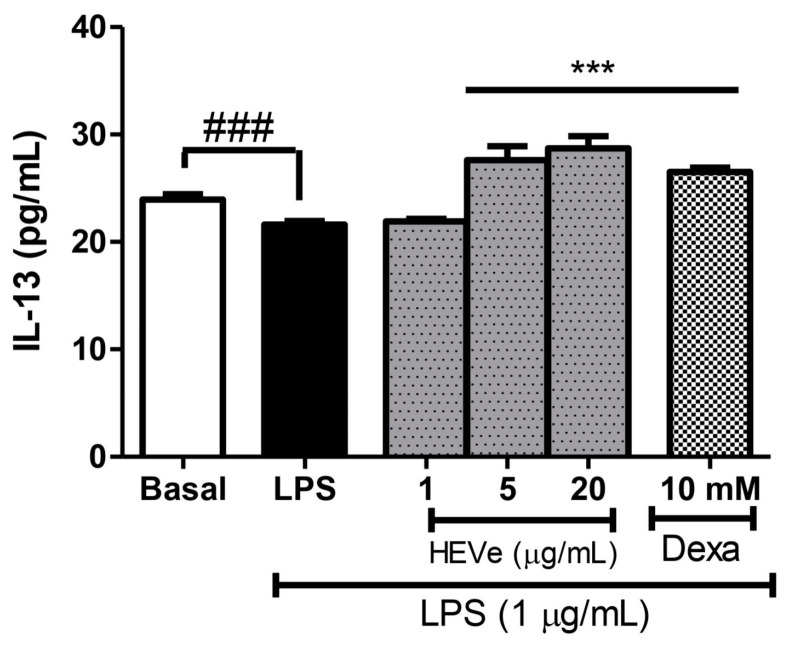
Effect of *in vitro* treatment with hydroethanolic extract from the inner stem bark of *Virola elongata* (HEVe 1, 5, and 20 µg/mL) and dexamethasone (Dexa 10 mM) on the concentration of the cytokine IL-13 in LPS (1 µg/mL)-stimulated RAW 264.7 cells. Cells in the basal group were treated only with DMEM culture medium with 10% fetal bovine serum (FBS). One-way ANOVA, followed by the Student–Newman–Keuls test, *** *p* < 0.001 vs. LPS, ^###^ *p* < 0.001 vs. Basal.

The determination of the cytokines in the RAW 264.7 cells induced by LPS (1 µg/mL) resulted in a reduction in the levels of the anti-inflammatory cytokine IL-13 by 9.8% (*p* < 0.001) compared to the basal group. The treatment with HEVe at 5 and 20 µg/mL increased IL-13 levels by 27.7% and 32.9%, respectively (*p* < 0.001). Dexamethasone (10 mM) increased IL-13 levels by 22.7% (*p* < 0.001) compared to the LPS group (Figure 9).

## 4. Discussion

The riverine community of Mato Grosso, a North Araguaia Microregion, utilizes an aqueous infusion or maceration of the dried stem bark of *V*. *elongata* in cachaça or white wine to treat inflammation and other illnesses [15]. To emulate the traditional method of use, in this study, an extract of *V*. *elongata* (HEVe) was prepared by macerating the inner stem bark in a hydroethanolic solution.

To assess the activity and anti-inflammatory mechanism of HEVe, this study employed *in vivo* experimental models of LPS-induced acute peritonitis and topical dermatitis induced by applying 2.5% croton oil to the ears of female mice, alongside *in vitro* assays using RAW 264.7 cells stimulated by LPS. Additionally, the phytochemical composition of the HEVe was determined using UHPLC-PDA-ESI-IT-MS analysis.

The mass spectrometry analysis revealed the presence of an important stilbene compound, resveratrol (3,4′,5-trihydroxystilbene), and a phenolic acid, quinic acid, which were identified in the UHPLC-MS chromatogram. Some of the anti-inflammatory effects of resveratrol are attributed to its ability to inhibit the production of IL-2 and interferon-gamma (IFN-γ) by lymphocytes and tumor necrosis factor-alpha (TNF-α), or of IL-12 by macrophages [32]. Quinic acid, previously identified [19], is a phenolic acid that inhibits vascular inflammation in vascular smooth muscle cells stimulated by TNF-α [33]. The UHPLC-MS also identified a compound from the lignan class (3′,4′-dimethoxy-3,4-methylenedioxy-6 7′,8 8′-neolignan). Some articles report that dietary lignans and their metabolites derived from the intestinal microbiota control the inflammatory response by suppressing inflammatory pathways, by blocking the expression of pro-inflammatory cytokines through the negative regulation of JAK/STAT, NF-κB, and AP-1 [34].

Flavonoids are among the most prevalent polyphenols found in plants and fruits. They are widely utilized to treat chronic inflammation and as dietary supplements [35]. According to Maleki et al. [36], flavonoids impact the metabolism of arachidonic acid by inhibiting PLA_2_, COX, and LOX enzymes, thus reducing the production of pro-inflammatory mediators such as PGs, TXs, and leukotrienes. Additionally, they modulate transcription factors like NF-κB, GATA-3, and STAT-6, leading to decreased transcription of pro-inflammatory genes.

The effect of HEVe on leukocyte recruitment was evaluated by inducing acute peritonitis in animals by intraperitoneal injection of the endoxin LPS, the main component in the membranes of Gram-negative bacteria [37]. The induction of peritonitis produces an inflammatory response in the peritoneal cavity, increasing the amount of existing proteins and the number of defense cells (leukocytes) in the exudate, with a predominance of polymorphonuclear cells [38,39].

Based on the literature, it is plausible that the anti-inflammatory properties of HEVe stem, at least in part, from the collective action of phenolic acids, neolignans, and flavonoids. Further studies are warranted to isolate these compounds and to conduct tests to ascertain whether they are active markers of HEVe or merely analytical indicators. In the experimental model of acute peritonitis, mice pre-treated with HEVe (5, 25, and 100 mg/kg) showed a reduction in the total number of leukocytes and polymorphonuclear neutrophils (PMNs), which are the major effectors of the acute inflammatory response [40]. The reduced presence of these cells in the peritoneal lavage indicates reduced cell migration, suggesting that the anti-inflammatory action of the extract involves this mechanism.

The inflammatory process involves the recruitment of certain cells to the site of inflammation, which generates a local response to combat the phlogistic agent [41]. PMNs are the first cells to migrate to the inflamed site, being the host’s initial defense against several pathogens [42] such as LPS, which can stimulate cells that secrete pro-inflammatory (e.g., TNF-α, IL-6, IL-1β and IFNɣ) and anti-inflammatory (e.g., IL-4, IL-10, IL-11, and IL-13) cytokines [43,44,45].

The quantification of cytokines provides valuable insights for monitoring patients’ immune system status and adjusting therapies for various inflammatory conditions [46]. We measured TNF-α, IL-1β, and IL-10 levels in peritoneal lavage to assess the involvement of cytokines in the anti-inflammatory effect of HEVe. The pretreatment with HEVe resulted in reduced levels of pro-inflammatory cytokines (TNF-α and IL-1β) and increased levels of the anti-inflammatory cytokine IL-10.

Pro-inflammatory cytokines such as TNF-α and IL-1β play crucial roles in immune responses, serving as vital mediators that communicate tissue distress due to infection or injury. Inhibiting excessive levels of these cytokines by HEVe can lead to reduced cellular activation, including macrophages, thereby modulating the immune response and, consequently, dampening the acute phase of inflammation [47].

A range of immunoregulatory molecules, including IL-10, are produced to mitigate the potentially detrimental effects of sustained or excessive pro-inflammatory reactions [46]. IL-10 functions by inhibiting the production of various pro-inflammatory cytokines, such as TNF-α, IL-1β, and IFN-ɣ, expressed by activated macrophages. Additionally, it promotes the proliferation of B cells and antibody production while suppressing cellular immunity and mast cell growth [45]. The increased levels of IL-10 induced by HEVe can mitigate inflammation progression by modulating the extent and duration of the inflammatory response [14,48]. After the HEVe’s anti-inflammatory effect on lipopolysaccharide-induced peritonitis was demonstrated, its local anti-inflammatory effect was evaluated using the topical dermatitis model, induced by the topical application of croton oil in the ears of female mice. Tetradecanoyl-phorbol acetate is a highly irritating substance derived from croton oil, which stimulates an inflammatory response in the epidermis through changes in the production of cytokines, adhesion molecules, prostaglandins, and leukotrienes [49]. The topical pre-treatment with HEgVe (1, 3, and 9%) promoted edema inhibition, which may be associated with the consequent reduction of inflammatory factors, such as vascular permeability, vasodilation, and swelling [50].

*In vitro* experimental tests were conducted on RAW 264.7 murine macrophage cells to assess whether the anti-inflammatory action of HEVe stems from a direct effect on inflammatory cells.

Murine macrophage cells have been widely utilized in studies due to their ease of cultivation and rapid growth [47], alongside their pivotal roles in the inflammatory response. Hence, preclinical evaluation in these cells is a crucial step in analyzing the efficacy of novel anti-inflammatory drug candidates, as the cells undergo various processes such as phagocytosis, reactive oxygen species (ROS) production, and cytokine secretion when stimulated by LPS [51,52].

RAW 264.7 cells stimulated with LPS (1 µg/mL) were pretreated with different concentrations of HEVe (6.25–400 µg/mL) to determine the appropriate concentrations of HEVe for the *in vitro* experiments, and cell viability was assessed using the Alamar Blue^®^ (resazurin) assay at 24, 48, and 72 h. Similarly to its non-cytotoxic effects in CHO-K1 cells at 24 and 72 h (IC_50_ > 200 μg/mL) [20], the HEVe exhibited an IC_50_ of 288 ± 19.56 μg/mL at 24 h in these RAW 264.7 studies. Consequently, concentrations of 1, 5, and 20 µg/mL were selected for further investigations of HEVe’s anti-inflammatory properties. Consistent with the *in vivo* assays, the pretreatment with HEVe also mitigated inflammatory effects in the RAW 264.7 cells by directly reducing levels of the pro-inflammatory cytokines IL-1β and TNF-α, while increasing levels of the anti-inflammatory cytokine IL-13. The reduction in excessive concentrations of pro-inflammatory cytokines in RAW 264.7 cells caused by HEVe may result from the direct suppression of specific cytokine pathways, preventing the development of pathological states through the exacerbated expression of immunological mediators [43]. The increase in IL-13 levels caused by HEVe favors the inhibition of inflammatory effects due to the reduction in the expression of vascular cell adhesion molecule (VCAM)-1 in endothelial cells, the proliferation of B cells, and the inhibition of many pro-inflammatory cytokines, inflammatory agents, and other mediators of inflammation (e.g., granulocyte-macrophage colony-stimulating factor (GM-CSF) PGs, reactive oxygen, and nitrogen intermediates) [43,53,54]. Based on these findings, it can be stated that the anti-inflammatory effect of HEVe results from changes in the balance of cytokine production.

Another crucial inflammatory mediator is NO, which, at normal levels, plays roles in maintaining low vascular tone, preventing leukocyte and platelet adhesion to the vascular wall, and neuromodulation. However, during the inflammatory process, the production of nitric oxide in macrophages is activated by the enzyme inducible nitric oxide synthase (iNOS), leading to the release of large amounts of NO, which can be toxic to the organism [55,56].

Due to its instability, NO rapidly converts to nitrate and nitrite [24]. Therefore, the indirect production of NO was determined by measuring NO_2_^−^ levels in RAW 264.7 cells stimulated by LPS. The treatment with HEVe (1, 5, and 20 µg/mL) resulted in a significant reduction in NO levels, which may be attributed to the consequent inhibition of iNOS. This reduction could mitigate effects such as tissue and endothelial damage, vascular permeability, and vasodilation [55,57]. These findings align with the results of Carvalho et al.’s study [58], which reported that the resin of Virola oleifera directly inhibited the LPS-induced production of NO in RAW 264.7 cells. Previous studies of HEVe reported gastroprotective and antioxidant activity, and identified its secondary metabolites as phenolic acid (gallic acid), stilbene (3,3,4-trihydroxystilbene), flavonoids (catechin and rutin), and neolignans (arylnaphthalene and dibenzylbutane) [19].

Phenolic compounds, such as gallic acid, exhibit anti-inflammatory and antioxidant activity [59]. Similarly, stilbenes, synthesized by plants like grapes, peanuts, rhubarb, and berries, serve as defense mechanisms against stressful conditions [60].

Corroborating the findings of Ribeiro et al. [18], which highlighted the traditional use of the macerated extract of *V. elongata*’s dried inner stem bark in cachaça for inflammation treatment, our *in vivo* and *in vitro* analyses of HEVe confirmed the anti-inflammatory activity of this species’ bark. The HEVe reduced the total leukocyte and PMN counts in the peritoneal cavities of the mice, partially inhibited the pro-inflammatory cytokines TNF-α and IL-1β, and increased the levels of the anti-inflammatory cytokine IL-10. Furthermore, in studies involving RAW 264.7 cells, HEVe exhibited low cytotoxicity, decreased nitric oxide and pro-inflammatory cytokine production, and elevated the levels of the anti-inflammatory cytokine IL-13. These results are likely to have been driven by the activity of HEVe’s secondary metabolites.

## 5. Conclusions

HEVe demonstrated oral and topical anti-inflammatory activity, validating the use of homemade preparations containing the stem bark of *V*. *elongata* for inflammation treatment. The multi-targeted anti-inflammatory action of HEVe is associated, at least in part, with leukocyte inhibition, cytokine balance modulation, and the inhibition of NO production. HEVe exhibited very low cytotoxicity, suggesting that its effectiveness stems from the presence of secondary metabolites belonging to the phenolic acid, stilbene, neolignan, and flavonoid classes. These findings provide scientific support for HEVe as a potential anti-inflammatory herbal medicine for clinical application.

## 6. Patents

“Composição fitoterápica obtida a partir do extrato seco da entrecasca do caule de *Virola elongata* (Benth.) Warb. e seu uso para o tratamento de inflamações” (process n. BR 10 2023 025511 6).—INPI (patent application requested).

## Figures and Tables

**Table 1:** Compounds identified through fragmentation pattern analysis obtained both by UHPLC-ESI-IT- and by DFI-ESI- IT-MS^n^ in the hydroethanolic extract of *Virola elongata* inner stem bark.

N°	Rt (min)	[M − H]^−^	MS/MS	Compound (Molecular Formula)	Reference
1	6.02	191	173, 127	Quinic acid (C_7_H_12_O_6_)	[19]
2	9.28	277	185, 183	Resveratrol (C_14_H_12_O_3_)	[29]
3	12.98	399	321, 295, 183	3′,4′-dimethoxy-3,4-methylenedioxy-6 7′,8 8′-neolignan (C_21_H_24_O_6_)	[19]
4	29.45	447	301	Quercetin (C_15_H_10_O_7_)	[30]
5	30.60	577	559, 451, 425, 407, 289	Catechin dimer (C_30_H_24_O_12_)	[31]
6	30.95	595	577, 505, 475, 415, 355	Diglycosyl-flavonoid (C-glycoside) (C_26_H_28_O_16_)	[19]
7	40.74	865		Catechin trimer (C_45_H_38_O_18_)	[31]
8	45.52	1153	576	Catechin tetramer (C_60_H_50_O_24_)	[31]
9	53.27	1441	720	Catechin pentamer (C_75_H_62_O_30_)	[31]

Almeida G. V. B. et al., (2019) [19], Moss et al. (2013) [29], Acquavia M. A. et al. (2021) [30], Prasain and Barnes (2014) [31].

## Data Availability

Data are contained within the article and Supplementary Materials.

## Supplementary Materials

The following supporting information can be downloaded at: https://www.mdpi.com/article/10.3390/biology13100776/s1, Figure S1. Interpretation of partial mass spectrometry results illustrating the fragmentation obtained in the analysis of Quinic acid (1) 191.0 Da. Referring to the high performance liquid chromatography analysis of the hydroethanolic extract of *Virola elongata*. Performed in negative mode. Almeida G. V. B. et al. (2019) [19]; Figure S2. Interpretation of partial mass spectrometry results illustrating the fragmentation obtained in the analysis of Resveratrol (2) (quinic acid 277.01 Da. Referring to the high performance liquid chromatography analysis of the hydroethanolic extract of *Virola elongata*. Performed in negative mode. Moss et al. (2013) [29]; Figure S3. Interpretation of partial mass spectrometry results illustrating the fragmentation obtained in the analysis of 3′,4′-dimethoxy-3,4-methylenedioxy-6 7′,8 8′-neolignan (3) 399.00 Da. Referring to the high performance liquid chromatography analysis of the hydroethanolic extract of *Virola elongata*. Performed in negative mode. Almeida G. V. B. et al. (2019) [19]; Figure S4. Interpretation of partial mass spectrometry results illustrating the fragmentation obtained in the analysis of Quercetin (4) 447.25 Da. Referring to the high performance liquid chromatography analysis of the hydroethanolic extract of *Virola elongata*. Performed in negative mode. Acquavia M. A. et al. (2021) [30]; Figure S5. Interpretation of partial mass spectrometry results illustrating the fragmentation obtained in the analysis of Catechin dimer (5) 425.10 Da. Referring to the high performance liquid chromatography analysis of the hydroethanolic extract of *Virola elongata*. Performed in negative mode. Barnes (2014) [31]; Figure S6. Interpretation of partial mass spectrometry results illustrating the fragmentation obtained in the analysis of Diglycosyl-flavonoid (C-glycoside) (6) 577.25 Da. Referring to the high performance liquid chromatography analysis of the hydroethanolic extract of *Virola elongata*. Performed in negative mode. Almeida G. V. B. et al. (2019) [19]; Figure S7. Interpretation of partial mass spectrometry results illustrating the fragmentation obtained in the analysis of Catechin trimer (7) 865.00 Da. Referring to the high performance liquid chromatography analysis of the hydroethanolic extract of *Virola elongata*. Performed in negative mode. Barnes (2014) [31]; Figure S8. Interpretation of partial mass spectrometry results illustrating the fragmentation obtained in the analysis of Catechin tetramer (8) 1153.02 Da. Referring to the high performance liquid chromatography analysis of the hydroethanolic extract of *Virola elongata*. Performed in negative mode. Barnes (2014) [31]; Figure S9. Interpretation of partial mass spectrometry results illustrating the fragmentation obtained in the analysis of Catechin pentamer (9) 1441.01 Da. Referring to the high performance liquid chromatography analysis of the hydroethanolic extract of *Virola elongata*. Performed in negative mode.
