# Supplementary material for: Phytochemistry and Evaluation of the Anti-Inflammatory Activity of the Hydroethanolic Extract of Virola elongata (Benth.) Warb. Stem Bark
Source: Biology (Basel). 2024 Sep 28;13(10):776. doi: 10.3390/biology13100776 (PMC11505066; doi:10.3390/biology13100776)
Supplement: Supplementary file 1 [file biology-13-00776-s001.zip › biology-3208081-supplementary.pdf]

Below is the supplementary supporting data from the MS for the identified specificities 1-9.

Supporting Supplementary Data

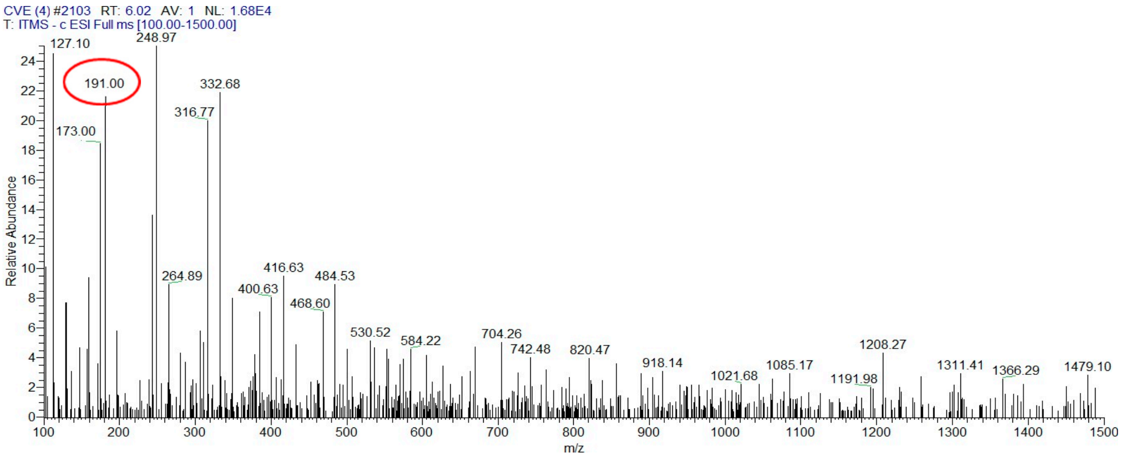

Figure S1. Interpretation of partial mass spectrometry results illustrating the fragmentation obtained in the analysis of Quinic acid (**1**) 191.0 Da. Referring to the high performance liquid chromatography analysis of the hydroethanolic extract of *Virola elongata.* Performed in negative mode. Almeida G. V. B. et al., (2019) [19]

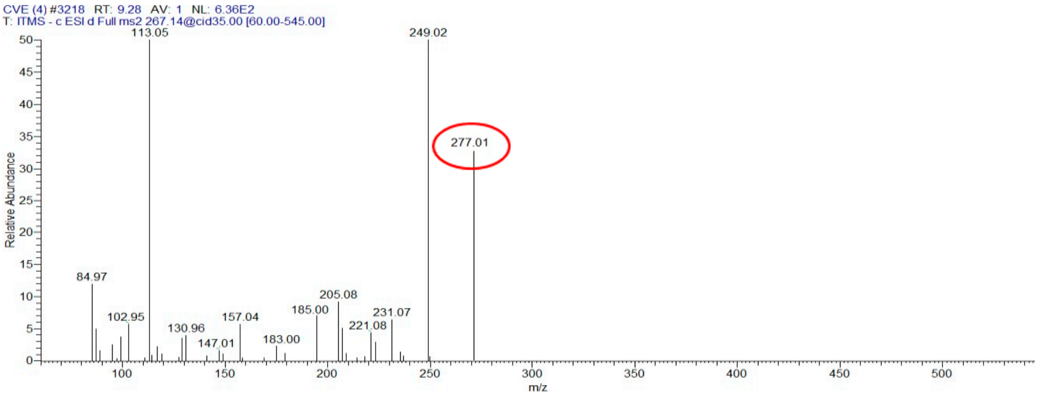

Figure S2. Interpretation of partial mass spectrometry results illustrating the fragmentation obtained in the analysis of Resveratrol (**2**) (quinic acid 277.01 Da. Referring to the high performance liquid chromatography analysis of the hydroethanolic extract of *Virola elongata.* Performed in negative mode. Moss et al. (2013) [29]

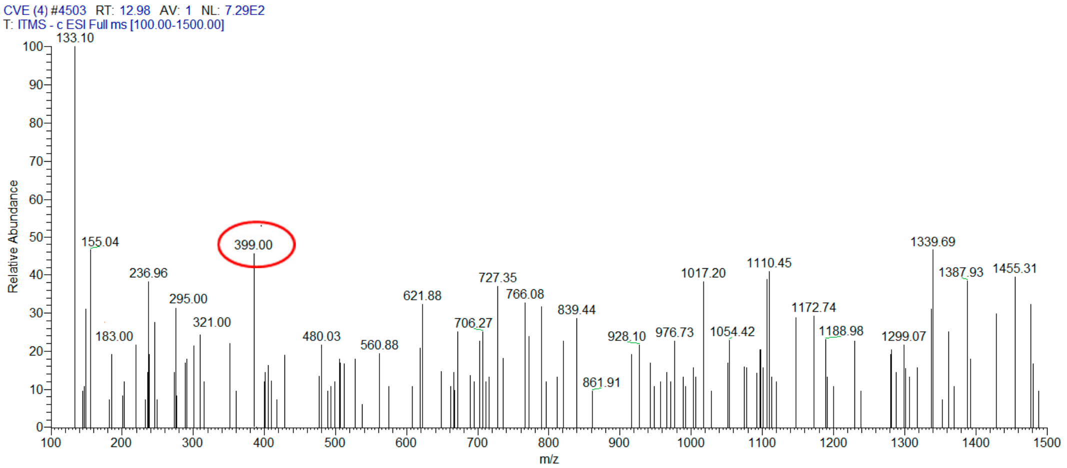

Figure S3. Interpretation of partial mass spectrometry results illustrating the fragmentation obtained in the analysis of 3′,4′-dimethoxy-3,4-methylenedioxy-6 7′,8 8′-neolignan (**3**) 399.00 Da. Referring to the high performance liquid chromatography analysis of the hydroethanolic extract of *Virola elongata.* Performed in negative mode. Almeida G. V. B. et al., (2019) [19]

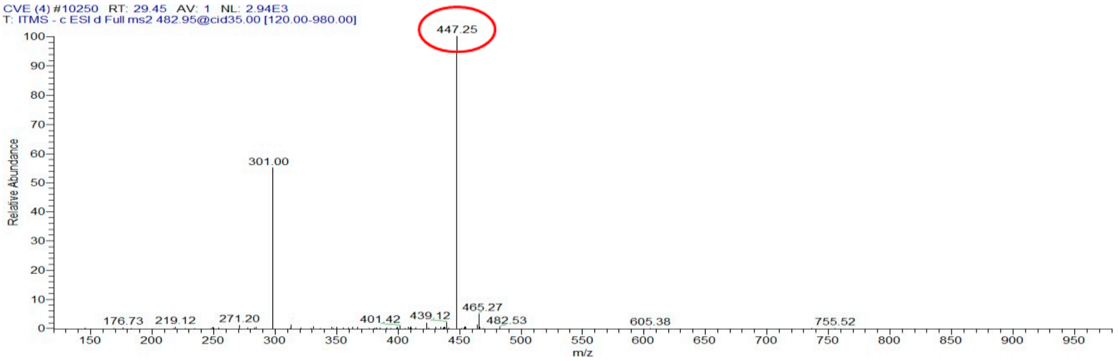

Figure S4. Interpretation of partial mass spectrometry results illustrating the fragmentation obtained in the analysis of Quercetin (**4**) 447.25 Da. Referring to the high performance liquid chromatography analysis of the hydroethanolic extract of *Virola elongata.* Performed in negative mode. Acquavia M. A. et al. (2021) [30]

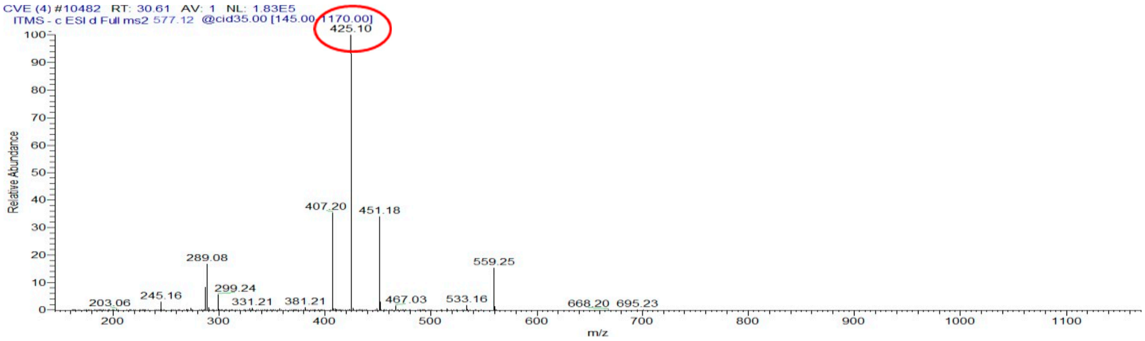

Figure S5. Interpretation of partial mass spectrometry results illustrating the fragmentation obtained in the analysis of Catechin dimer (**5**) 425.10 Da. Referring to the high performance liquid chromatography analysis of the hydroethanolic extract of *Virola elongata.* Performed in negative mode. Barnes (2014) [31]

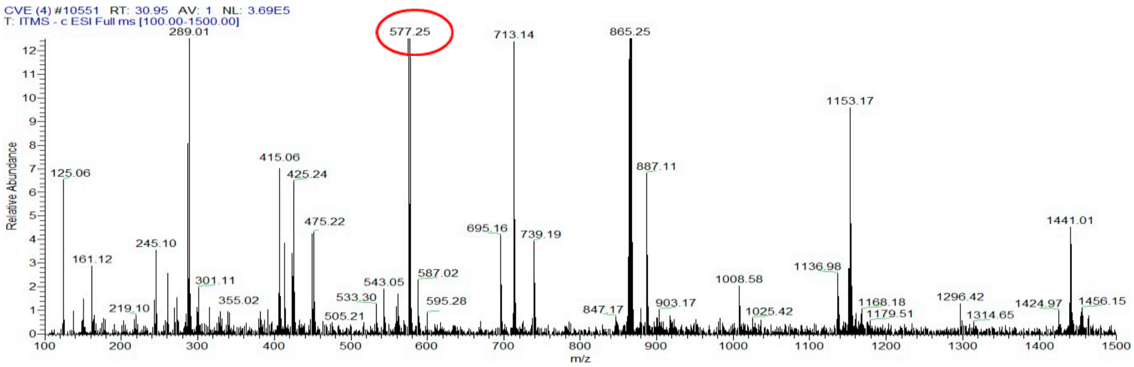

Figure S6. Interpretation of partial mass spectrometry results illustrating the fragmentation obtained in the analysis of Diglycosyl-flavonoid (C-glycoside) (**6**) 577.25 Da. Referring to the high performance liquid chromatography analysis of the hydroethanolic extract of *Virola elongata.* Performed in negative mode. Almeida G. V. B. et al., (2019) [19]

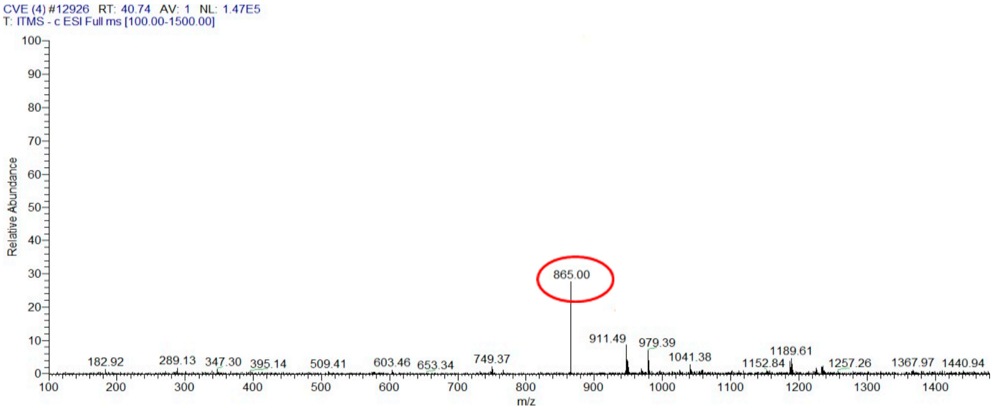

Figure S7. Interpretation of partial mass spectrometry results illustrating the fragmentation obtained in the analysis of Catechin trimer (**7**) 865.00 Da. Referring to the high performance liquid chromatography analysis of the hydroethanolic extract of *Virola elongata.* Performed in negative mode. Barnes (2014) [31]

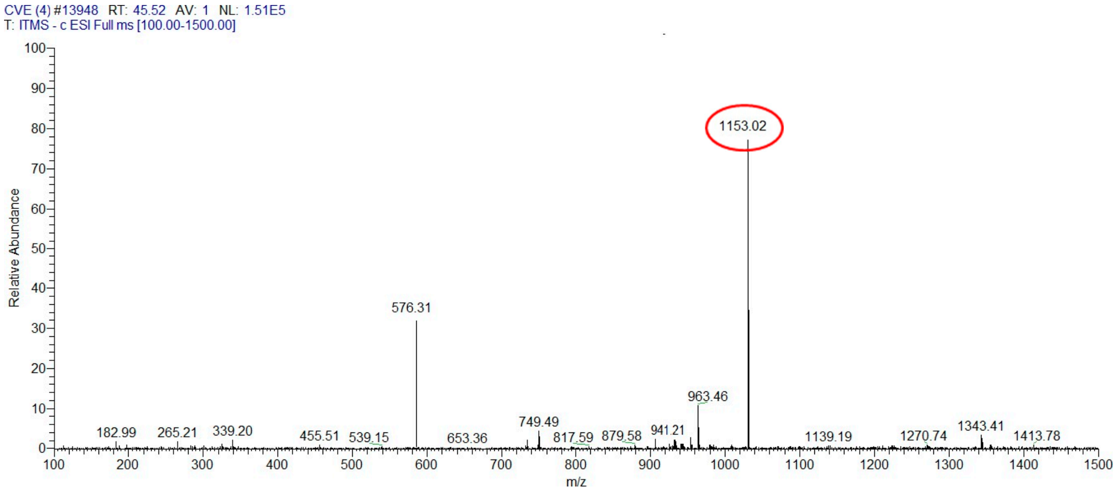

Figure S8. Interpretation of partial mass spectrometry results illustrating the fragmentation obtained in the analysis of Catechin tetramer (**8**) 1153.02 Da. Referring to the high performance liquid chromatography analysis of the hydroethanolic extract of *Virola elongata.* Performed in negative mode. Barnes (2014) [31].

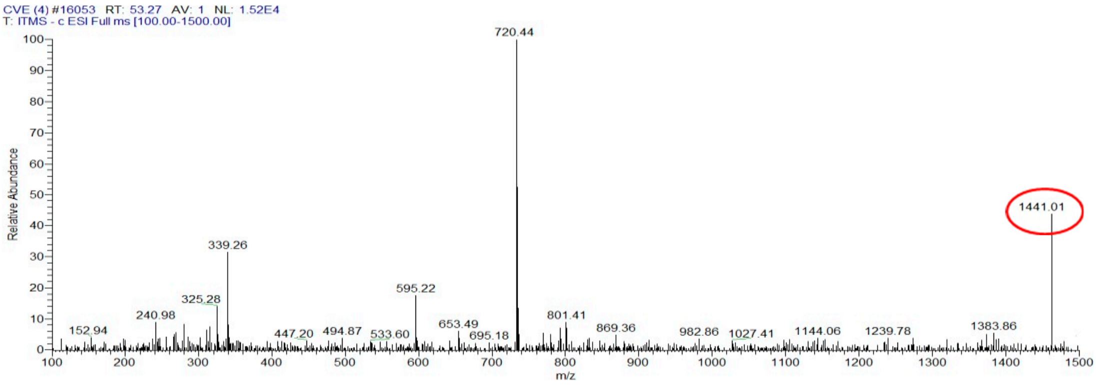

Figure S9. Interpretation of partial mass spectrometry results illustrating the fragmentation obtained in the analysis of Catechin pentamer (**9**) 1441.01 Da. Referring to the high performance liquid chromatography analysis of the hydroethanolic extract of *Virola elongata.* Performed in negative mode.
